# The epidemiology of tick in transmission of *Enterobacteriaceae* bacteria in buffaloes in Marshes of the south of Iraq

**DOI:** 10.14202/vetworld.2018.1677-1681

**Published:** 2018-12-16

**Authors:** Jenan Mahmood Khalaf, Ibrahim Abbas Mohammed, Abdulkarim Jafar Karim

**Affiliations:** Department of Internal and Preventive Medicine, College of Veterinary Medicine, University of Baghdad, Baghdad, Iraq

**Keywords:** epidemiology, ticks, *Enterobacteriaceae*, buffaloes, Iraq

## Abstract

**Aim:**

This study aimed to investigate the role of ticks in transmission of *Enterobacteriaceae* bacteria in buffaloes in marshes of the south of Iraq.

**Materials and Methods:**

This survey included 255 healthy and clinically ill buffaloes in marshes of the south of Iraq (Thi-Qar, Basra, and Misan provinces) between the periods from May 2017 to April 2018. Animals were clinically examined. Ticks, isolated from perineum and under tail, sent to the Department of Parasitology, College of Veterinary Medicine, University of Baghdad and University of Thi-Qar for taxonomy. Ticks were dissected, and all internal organs were removed aseptically by forceps to sterile tubes containing brain heart infusion broth and incubated at 37°C for 36 h and subcultured on blood and MacConkey agars at 37°C for 36 h. Biochemical tests including citrate, methyl red, indole, urease, triple sugar iron (H_2_S), motility tests, and Gram stain were performed.

**Results:**

Two species of ticks were identified. *Hyalomma* spp. (175; 68.63%) were significantly higher than *Rhipicephalus* spp. (80; 31.37%). Conversely, pathogenic bacteria in *Rhipicephalus* spp. (55; 68.75%) was higher than detected from *Hyalomma* spp. (113; 64.57%), but non-significant. The prevalence of *Enterobacteriaceae* bacteria in ticks on diseased buffaloes (110; 88.00%) was significantly higher than non-diseased (58; 44.61%). *Escherichia coli* (123; 73.21%) showed a significantly higher prevalence than *Salmonella* spp. (25; 14.88%) and *Klebsiella* spp. (15; 8.92%). There was no significant variation between *Salmonell*a spp. and *Klebsiell*a spp. The latter was significantly higher than *Enterobacter* spp. (5; 2.97%). The isolation rate of infected tick collected from buffaloes inhabiting marshes was 65 (66.32%), 45 (69.23%), and 58 (63.40%) from Thi-Qar, Basra, and Misan provinces, respectively, with no significant variation. July and August (71.05% and 72.97%) reported the highest among months, while November, December, January, and February recorded nil (0.00%). The summer season was significantly higher (72.72%) followed by autumn (62.06%) and spring (59.77%), while winter reported no any bacterial isolation (0.00%).

**Conclusion:**

The high prevalence of *Enterobacteriaceae* bacteria isolated from hard ticks supports the probability of transmitting these bacteria to buffaloes in marshes of the south of Iraq.

## Introduction

Mesopotamian marshlands’ buffaloes distribute mainly in Iraq in three southern provinces, Thi-Qar, Misan, and Basra, with high population, primarily for milk production and for meat [1]. Hard ticks act as mechanical vector to pathogenic microorganisms, and they are incriminated in significant economic losses [2]. In addition to tissue damage, irritation, hypersensitivity, abscess, and anemia, ticks may reduce productivity when present in large number [3,4]. Ticks are the main vector for protozoal diseases such as babesiosis, theileriosis, anaplasmosis [5], and flavivirus which cause tick-borne encephalitis [6]. Hard tick can also carry different pathogenic agents and cause serious human diseases [7], for instances, *Rickettsiosis*, Lyme disease, boutonneuse fever, Rocky Mountain spotted fever, Q-fever, and Crimean-Congo hemorrhagic fever [8] and several bacterial pathogens such as *Escherichia coli*, *Salmonella* spp., *Klebsiella* spp., *Serratia* spp., *Shigella* spp., and *Enterobacter aerogenes*, *Pasteurella multocida*, *Brucella abortus*, and *Salmonella typhimurium* in man and animals [9-11].

Tick-borne diseases represent a major zoonotic hazard worldwide. High prevalence of ticks infected with pathogenic microorganisms has been observed in urban and rural areas, worldwide [12-14]. *Hyalomma* and *Rhipicephalus* are common genera in Iraq, Middle East, and another part of Asia, Europe, and Africa. These hard ticks are of medical, veterinary, and economic importance because they are the vector of a lot of these pathogens [9,15-17].

The present study aimed to isolate *Enterobacteriaceae* bacteria from hard ticks that infect buffaloes in marshes of the south of Iraq.

## Materials and Methods

### Ethical approval

All tests and procedures were approved by the Animal Care and Use Committee in the College of Veterinary Medicine, University of Baghdad, approval no. 1752, on 28 April 2017.

### Hard tick collection

**Figure-1:**
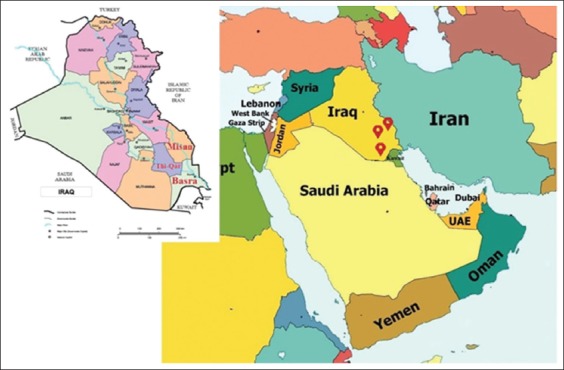
Locations of the study performed on buffaloes in marshes of the south of Iraq.

The survey study on hard tick isolated from buffaloes was carried in different locations in the marshes of the southern provinces of Iraq (Thi-Qar, Basra, and Misan) between the periods from May 2017 to April 2018 (Figure-1). 255 buffaloes were examined clinically, some buffaloes showed illness clinically, and others were clinically healthy. Ticks were isolated by forceps from perineum, under tail, and from udder and kept in test tube containing 70% alcohol [18]. Samples were sent for taxonomy to the Department of Parasitology, College of Veterinary Medicine, University of Baghdad and University of Thi-Qar. Ticks were morphologically identified using standard taxonomic keys according to Estrada-Pena *et al*. [19] by means of a stereomicroscope (Leica MZ16^®^, Germany).

### Bacterial culturing

Ticks were dissected according to Edward *et al*. [20] by embedding ticks in paraffin, removing tick integument, then, aseptically removing all the internal organs by forceps to sterile tubes containing brain heart infusion broth. The tubes were incubated at 37°C for 36 h, then cultured on blood agar and incubated at 37°C for another 24 h for another time and then subcultured on MacConkey agar at 37°C for 36 h. Biochemical tests including citrate, methyl red, indole, urease, and triple sugar iron (H_2_S) tests, in addition to Gram stain, were performed according to Quinn *et al*. [21].

### Statistical analysis

Data were analyzed by SPSS program (version 9.1, 2010) using Chi-square test for comparison at statistically significant level of p≤0.05 [22].

## Results and Discussion

Tick-borne diseases remain acting as a silent war with increasing causalities. Recently, several fatal cases of Crimean-Congo hemorrhagic fever (CCHF) occurred in Iraq [23] may be transported across borders with Turkey [24,25]. The role of *Hyalomma* and *Rhipicephalus* ticks in maintaining the viruses such as CCHF, West Nile virus, yellow fever, and Japanese encephalitis, and many other bacterial diseases in endemic foci is possibly flare up at any moment [6,12,26,27]. Humans become infected from a tick bite or by direct contact with animals bitten by infected ticks. *Hyalomma* and *Rhipicephalus* are the most efficient and common vectors for transmitting diseases [12].

### Distribution of the general genus of hard ticks

A total of 255 hard ticks were isolated; 175(68.63%) for *Hyalomma* spp. and 80 (31.37%) for *Rhipicephalus* spp. (Table-1). *Hyalomma* spp. were significantly higher than *Rhipicephalus* spp., although non-significant (p≤0.05) increment of the pathogenic bacteria in *Rhipicephalus* spp. (55; 68.75%) than *Hyalomma* spp. (113; 64.57%) was reported (Table-1). These two genera are common in Iraq and many other parts of Asia, Europe, and Africa [9,14]. Both are distributed in hot area, but *Hyalomma* spp. prefer wet area [28] while *Rhipicephalus* spp. are common in dry area [29]. In Turkey, among 10 different genera detected, the incidence of *Hyalomma* spp. and *Rhipicephalus* spp. was 67.01% and 11.43%, respectively, widely differed from our results [14]. This variation may be attributed to the wide climatic diversity between Turkey and Iraq. Our result agreed with Akhtar *et al*. [28], Mohammad and Jassim [30], and Kirecci *et al*. [11] who argued that both *Hyalomma* spp. and *Rhipicephalus* spp. cohabit the same animal in the same environment, and therefore, no significant variation in the incidence of isolated bacteria from these two genera was obtained.

**Table-1:** Prevalence of isolated hard ticks in relation to bacterial infection.

Genus	Samples (%)	Positive bacteria (%)
*Rhipicephalus* spp.	80 (31.37)	55 (68.75)
*Hyalomma* spp.	175 (68.63) *	113 (64.57)
Total	255	168 (65.88)

* Refers to significant variation at p≤0.05

### Prevalence according to clinical signs

The occurrence of ticks according to clinical signs in accordance with pathogenic agents peaked to 110 (88.00%) in diseased buffaloes (Table-2), significantly higher (p≤0.05) than the non-diseased (58; 44.61%).

**Table-2:** Prevalence of ticks infected with *Enterobacteriaceae* bacteria from buffaloes according to clinical signs.

Clinically	Samples	Positive bacteria	Prevalence (%)
Diseased	125	110	88.00*
Non-diseased	130	58	44.61
Total	255	168	65.88

* Refers to significant variation at p≤0.05

### Prevalence of pathogenic Enterobacteriaceae

**Figure-2:**
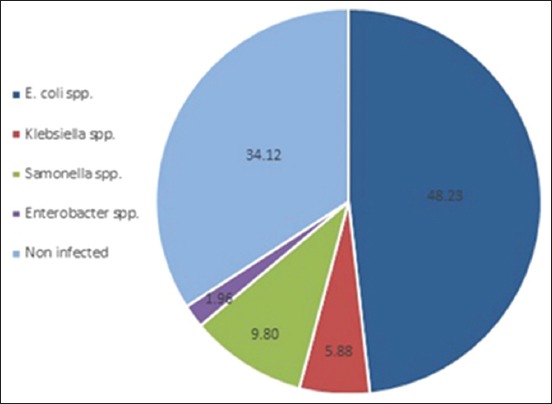
Prevalence of *Enterobacteriaceae* bacteria isolated from hard ticks infested on buffaloes.

Pathogenic bacteria from *Enterobacteriaceae* family (168; 65.88%) were isolated from hard ticks including *E. coli* (123; 48.03%), *Salmonella* spp. (25; 9.8%), *Klebsiella* spp. (15; 5.88%), and *Enterobacter* spp. (5; 1.96%). Significant increment (p≤0.05) was reported in the occurrence of *E. coli* from all other species (Figure-2). The isolation and identification followed the shape of growth on blood agar and MacConkey agar and biochemical tests were as described by Quinn *et al*. [21]. In general, *E*. *coli* and *Enterobacter* spp. were motile and differed from the non-motile *Klebsiella* spp. and *Salmonella spp. E. coli* specified in indole production, while only *Salmonella* spp. produced H_2_S. *E. coli* and *Salmonella* spp. were positive to methyl red and negative to Voges–Proskauer, in contrast to *Klebsiella* spp. and *Enterobacter* spp. Finally, *Klebsiella* spp. and *Enterobacter* spp. were positive to citrate utilization and urease, *E. coli* was negative for both, and *Salmonella* spp. utilized citrate and negative for urease. Our study revealed that the prevalence of pathogenic agents in diseased buffaloes was significantly higher than in non-diseased. This result coincided with Jalil and Zenad [31]. Ultimately, ticks role as a mechanical vector to pathogenic microorganisms may prone animals for many life-threatening diseases and reducing animal productivity [2-4,32,33].

Our study agreed with Al-Amura and Almyahii [10], Jalil [17], Jalil and Zenad [31], and Dietrich *et al*. [34] who argued that the most commonly isolated bacteria were *E. coli*, a significantly higher than other agents. Our records were very close to that reported by Kirecci *et al*. [11] who found *E. coli* (62.5%), *Salmonella* spp. (10.97%), *Klebsiella* spp. (6.1%), and *Enterobacter* spp. (1.2%). Both Al-Amura and Almyahii [10], and Jalil and Zenad [31] could not isolate any *Salmonella* spp. from their samples, while the first team failed to isolate *Enterobacter* spp., and the second team failed to isolate *Klebsiella* spp. Although Karasartova *et al*. [14] recorded an infection rate of 100% in *Dermacentor* spp., 89% in *Haemaphysalis* spp., and 75% in *Ixodes* spp., the infection rate in *Hyalomma* spp. and *Rhipicephalus* spp. was 37% and 27%, respectively, significantly lower than our findings (Table-1). They isolated 17 microorganism species without referring to *Enterobacteriaceae* bacteria. Nevertheless, our total infection rate of ticks (65.69%) corresponds only to *Enterobacteriaceae* genus; this might be exaggerated to 100% infection rate, if we investigated bacteria other than *Enterobacteriaceae*, *Rickettsia*, protozoa, and viruses [14].

Such redundancy in *Enterobacteriaceae* isolates and their wide prevalence in marshes nature and the extensive fecal contamination among buffaloes area might probably increase ticks to harbor such pathogens. Therefore, the increased isolation rate of *Enterobacteriaceae* genus referred to unhygienic sanitation and bad management and ultimately increased the environment pollution through these two tick species. Some researchers showed similar isolates, whereas others showed different bacterial isolates according to various regions and times [11,34].

### Prevalence of tick infected pathogenic agents according to provinces, months, and seasons

The prevalence of tick infected with pathogenic agents was 65 (66.32%), 45 (69.23%), and 58 (63.4%) from buffaloes settled in Thi-Qar, Basra, and Misan marshes, respectively, with no significant variation. August showed the higher incidence (72.97%) among months, while no any tick was collected in November, December, January, and February (Table-3). On the other hand, summer season was significantly higher (72.72%) than Autumn (62.06%) and Spring (59.77%). All seasons showed significant differences compared with winter (Table-3). Increased temperature and humidity lead to increase the reproductive activity of ticks [35,36]. This fact coincided our significant finding of the occurrence of ticks in hot seasons, a highly suitable environment for tick reproduction, explaining ticks coming out of their slumber and search for suitable hosts [31]. Ticks may take infection from the environment of marshes or contaminated hair or skin of the buffaloes. Simultaneously, this period is the optimal environment for multiplication and growth of bacteria. Our finding agreed with Das [37], Hadi and Fotohi [38], and Salim *et al*. [16] who found that the prevalence of infected ticks in summer is higher than other months.

**Table-3:** Number of ticks infected with bacteria according to months and seasons.

Month (2017–2018)	Season (%)	Samples	Positive	Prevalence (%)
June	Summer (72.72)*	35	23	65.71*
July		38	30	71.05*
August		37	27	72.97*
September	Autumn (62.06)*	30	19	63.33*
October		28	17	60.71*
November		0	0	0
December	Winter 0	0	0	0
January		0	0	0
February		0	0	0
March	Spring (59.77)*	27	15	55.55*
April		30	17	56.66*
May		30	20	66.67*
Total		255	168	65.88

* Refers to significant variation at p≤0.05

## Conclusion

The high prevalence of *Enterobacteriaceae* bacteria isolated from hard ticks supports the probability of transmitting these bacteria to buffaloes in marshes of the south of Iraq. The authors recommend a good sanitary precaution and elimination of ticks to avoid the flare up of serious bacterial diseases.

## Authors’ Contributions

JMK designed the experiment. IAM and JMK interpreted the data and drafted the manuscript. IAM carried out the experiment. JMK and AJK performed the statistics and interpreted the data. AJK and JMK edited the manuscript. All authors read and approved the final manuscript.
